# The role of Rayleigh anomalies in the coupling process of plasmonic gratings and the control of the emission properties of organic molecules

**DOI:** 10.1038/s41598-022-07216-1

**Published:** 2022-02-25

**Authors:** Sarah Hamdad, Amadou T. Diallo, Mahmoud Chakaroun, Azzedine Boudrioua

**Affiliations:** Laboratoire Physique des Lasers, CNRS (UMR7538), Université Sorbonne Paris Nord, Sorbonne Paris Cité, 93430 Villetaneuse, France

**Keywords:** Engineering, Nanoscience and technology, Optics and photonics, Physics

## Abstract

We report the investigation of the influence of periodic metallic arrays on the emission properties of organic emitters. Beforehand, the study of the coupling process between nanoparticles through the analysis of the extinction spectra related to Rayleigh anomalies indicate the crucial role of those latter in defining the nature of the excited grating modes. The obtained results emphasis that Rayleigh Anomalies can be considered as the intermediate between individual plasmonic and collective photonic responses. Thereafter, the experimental and numerical studies of the lattice modes and their associated effects on the lifetime and emission directivity of nearby emitters indicate that tuning the geometrical grating parameters offers a possibility to select a particular coupling process from a localized effect to a far field response. Depending on the coupling strength, the emission can be strongly altered by increasing the density of states or providing diffractive orders. Eventually, this study reports that the Rayleigh Anomalies play the role of an excitation source which drives the nanoparticles to act as a set of diffractive objects for shaping the emission to be highly directive.

## Introduction

Over several years, a high attention has been given to coupled metallic nanoparticles (NPs) in one (1D) and two-dimensional (2D) arrays^1,2^. Several studies have been reported on their collective mode properties^3–6^. Specially, many works have shown the possible high-energy confinement and electric field enhancement using plasmonic arrays^7–9^. The excited modes are sensitive to several parameters, such as the polarization and the angle of the incident light, the geometrical grating parameters, and the refractive indices of the surrounding media. Besides, the interactions between NPs can have an important impact on the performances of optical devices where they are used. In the particular case of light sources, these interactions are crucial as NP gratings drastically influence the emission features of nearby emitters. Indeed, depending on several coupling parameters, the plasmonic effects^10^ can enhance or quench the emission of active molecules. Nanoparticle arrays exhibit also Rayleigh anomalies^11^, which are associated to a diffractive phenomenon at a grazing angle in the plane of the structure. They appear on the extinction spectra as sharp peaks, and particularly depend on the angle of the incident wave, the index of the surrounding medium and the grating period^11,12^. Physically, they are due to the appearance or disappearance of a diffraction order, which corresponds to a modification from an evanescent to a propagating mode and vice versa.

It has been shown that for short grating periods, when the spectral detuning between the localized surface plasmon resonance (LSPR) and the Rayleigh anomalies (λ_RA_) is large, the grating exhibits only localized plasmonic feature. However, when the lattice constant is chosen comparable to λ_RA_, the structures sustain the so-called surface lattice resonances (SLR), mixed modes sharing both plasmonic (LSPR) and photonic properties^13^. These SLR are long living modes, delocalized on several grating cells. They appear as narrow resonances with high quality factors indicating the low losses experienced by these hybrid states. The presence of the Rayleigh Anomalies in the structure appears to be a necessary condition to excite the SLR modes. At these specific wavelengths, NPs participate to collective diffraction phenomena where the energy is distributed in the plane of the array.

Actually, two regimes have been distinguished^14^: purely plasmonic one in which the mode intensity is confined near the nanoparticle and a hybrid one, in which the surface mode propagates over the structure resulting from a coherent scattering of light in the array. These excited SLR modes have been described as mixed modes of localized plasmons vibrations combined with diffracted grazing waves^15^.

Furthermore, the lattice mode locally affects the surrounding medium and leads to a modification of the free space properties. For instance, several studies showed the impact of metallic nanoparticles on the spontaneous emission rates of emitters placed in their vicinity^16–18^: the excitation of surface plasmons can provide new channels for emitters to decay^19,20^. Substantial enhancement of the decay rate of fluorescent dye molecules nearby nano-antenna structures as well as spectral changes have been reported^14,21^. Moreover, the presence of ordered metallic nano-structures can change the directivity of the emitted power^22,23^. For instance, periodic metallic set of shallow grooves were found to modify the emission directivity of fluorescent molecules; and, depending on the aperture size relative to the surface plasmon wavelength, the emission can be enhanced or cancelled in well-defined directions.

However, although, the literature is quite extensive on this subject, there are still many aspects to study and to thoroughly understand. For instance, a deep understanding of the coupling mechanisms between NPs is still required. The impact of varying the inter-particle strengths, the evolution from near to far field interactions, and the origin of the transition from a purely plasmonic to the regime dominated by diffractive effects has not been clearly defined. The control of these phenomena represents an efficient tool to select a particular optical response in the near or far field zone^24^, which in turn greatly influences the emission properties of nearby emitters. More specifically, the role of the Rayleigh Anomalies (RA) as key features and their impacts on the grating responses should be clearly understood. Several works on this subject continue to provide debate and controversy.

In this context, the aim of our work is to study the role of Rayleigh Anomalies (RA) in the coupling process between Ag-NPs in periodic grating and their influence on the optical properties of nearby organic molecules. The first part of this report deals with the study of Ag square array properties as a function of inter-particle distance. We numerically and experimentally study different lattice regimes by varying the grating period. We particularly evidence the optical features associated to the localized and far field effects and analyse the origin of the transition that leads to the generation of hybrid responses. The second part focuses on the coupling between NP gratings and organic emitters. We study the coupled NP-emitter system under optical pumping regime by considering various periods of Ag arrays. Lifetime measurements and emission pattern characterizations have been performed. Moreover, the spatial evolutions of the diffraction orders associated to the arrays have been, experimentally and theoretically, studied. In fact, to the best to our knowledge, the spatial evolutions of the delocalized modes have not been reported yet, in the case of metallic gratings.

## Results and discussion

### Metallic nanoparticle gratings

The typical device studied consists of a substrate of glass covered by a thin layer of an indium-tin oxide (ITO) of 140 nm thickness, on which a square array of silver nano-cylinders, with a diameter of 100 nm and a height of 35 nm, spatially extended on an area of 50 × 50 μm, is fabricated and uniformly covered by a passive thin organic layer of 100 nm 4,4′,4″-Tris[phenyl(m-tolyl)amino]triphenylamine (m-MTDATA).

**Figure 1:**
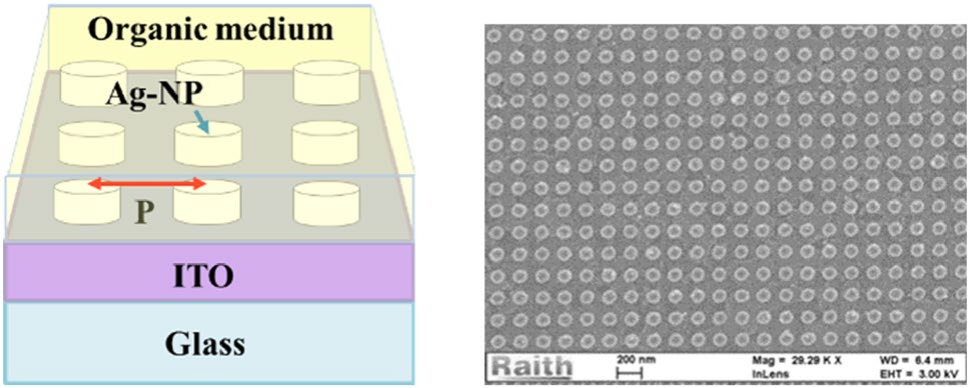
The studied sample and a SEM image of the realized Ag nanoparticle grating at a period of 280 nm.

The periodic structures have been realized using electronic beam lithography technique (*EBL*). The parameters are chosen in order to obtain grating resonances situated in the visible spectrum; wavelengths of interest as they match the emission of the most known organic active media. It is worth noting that the use of silver is related to its lowest optical damping properties in the visible spectrum compared to the other noble metals^25^ and, the possible tuning of the resonance at the wavelength of interest. Figure 1 displays a general scheme of the studied nanostructures as well as an SEM image of 280 nm-Ag square lattice.

**Figure 2:**
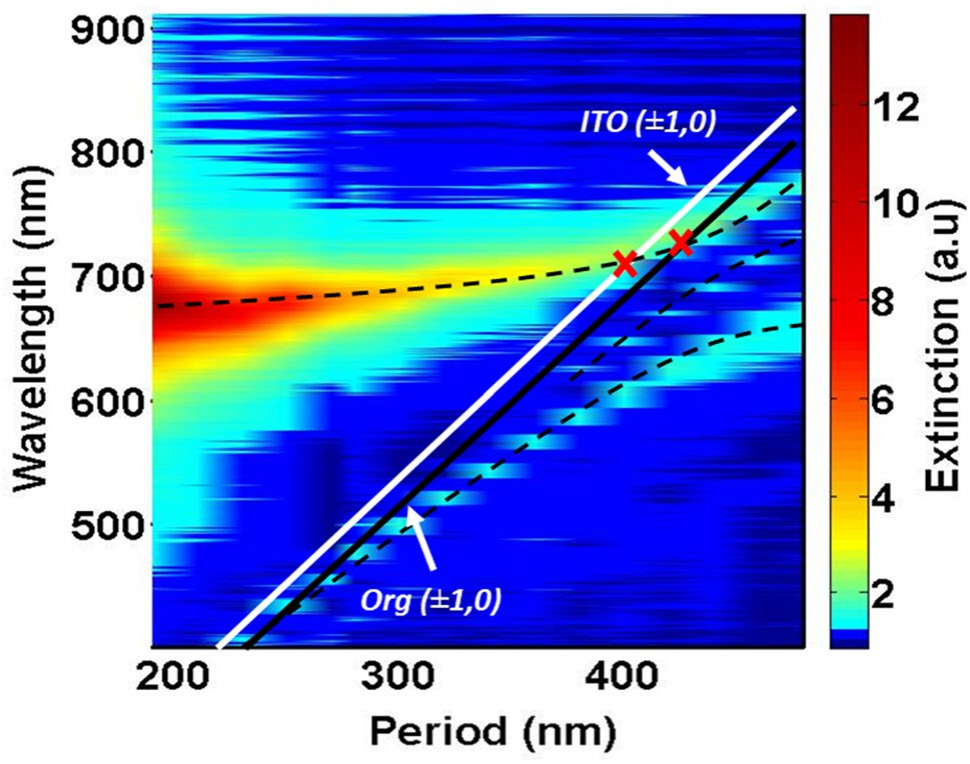
Extinction map intensity for Ag nanocylinders with a diameter of 100 nm as a function of the period and wavelength, and the corresponding (± 1, 0) Rayleigh anomalies orders in the media of the structure. The color bar on the right represents the extinction intensity.

The optical extinction responses of the gratings have been studied by varying the inter-particle distance $p$ from 200 to 480 nm by a step of 20 nm , under a normal incidence impinging from the substrate side. Figure 2 presents a map of the experimental extinction spectra related to these gratings, obtained by using a supercontinuum light source for the excitation and an Ocean Optics $2000 +$ for the spectral analysis. We mainly observe two principal branches. In order to analyze these results, we also report the ($\pm$ 1, 0) Rayleigh anomalies (equivalent to the (0, $\pm$ 1) orders) associated to the ITO substrate and the organic layer, which are associated to a diffractive phenomenon at grazing angles in the plane of the periodic array. In the particular case of a square array under a normal excitation, these Rayleigh anomalies are given by the positions of $\lambda_{RA}$ following the equation^11,12^:

1 $$ \lambda_{RA} = \frac{p}{{\sqrt {n^{2} + m^{2} } }}n_{d} $$

$$p $$ is the grating constant,$$ n_{d}$$ the refractive index of the medium where the diffraction occurs and $$n$$ and $$m$$ are integers which define the diffraction order.

It should be noted that we observe crossing points between the upper branch and the RA_ITO_ ($\pm$ 1.0) and RA_org_ ($\pm$ 1.0) at $p = 380\,{\text{nm}}$ and $p = 430\,{\text{nm}}$ respectively, which will be discussed below.

For a better insight of the grating behavior, let us consider three main zones following the array period: $p \le 280\,{\text{nm}}$, $280\,{\text{nm}} < p \le 430\,{\text{nm}}$ and $p > 430\,{\text{nm}}$.

For the periods $p < 280\,{\text{nm}}$, the upper branch (UB) shows a well-defined broad resonance peak at a wavelength of $670\,{\text{nm}}$. However, this peak is red shifted and becomes narrow when the period increases above $280\,{\text{nm}}$. As it will be stated below, we sustain that this branch gives the evolution of the resonance peak from a purely LSPR to an SLR one. Whereas, the lower principal branch (LB) varies in the reverse way, i.e. from an SLR to an LSPR peak. We notice that the beginning of the SLR-LB branch, for $p < 280\,{\text{nm}},$ completely merges with the Rayleigh anomaly in the organic medium RA_org_ ($\pm$ 1.0). It is worth noting that for $p < 280\,{\text{nm}}$, the extinction spectra exhibit a broad peak with a FWHM (Full Width at Half Maximum) of nearly $49\,{\text{nm}}$ at a wavelength of $\lambda_{LSPR} =$ 670 nm, corresponding to the dipolar LSPR of an isolated Ag-NP. In this zone, the spectral detuning between the LSPR and the RA_org_ ($\pm$ 1.0) is large leading to a dominant plasmonic responses for these arrays.

As the inter-particle distance increases, for $280\,{\text{nm}} < p \le 430\,{\text{nm}}$, the LSPR mode is strongly modified with a significant shift to the red range of the visible spectrum and an important reduction of the FWHM to $22.6\,{\text{nm}}$. In addition to that, the width of the SLR-LB branch increases from 9 to $16.6\,{\text{nm}}$, and its spectral detuning from the RA_org_ ($\pm$ 1.0) becomes more pronounced. In fact, up to $p = 430\,{\text{nm}}$, we observe a strong localization of the mode associated to this branch. Besides, in this period range, we can clearly observe the appearance of a secondary branch as the inter-particle distance increases. As shown on the extinction spectra, the intensity of the related peak is too weak to be observed on the map. This peak is strongly intensified within the zone of the anti-crossing between the two principal branches. This effect can be attributed to a partial coupling of the LSPR to the RA excited in the ITO medium. In fact, we can observe on the extinction map that this effect appear when the dispersion of the RA_ITO_ ($\pm$ 1.0) cross the main LSPR branch (the first crossing point on the map) at nearly $380\,{\text{nm}}$.

These behaviors of the branches suggest an effective energy transfer from the LSPR mode to the narrow resonance peaks in the presence of RA_org_ ($\pm$ 1.0) and RA_ITO_ ($\pm$ 1.0). These Rayleigh anomalies appear to play the same role of coupling the NPs in the gratings, which leads to the excitation of the two SLR modes, in the organic and the ITO media, respectively. However, as shown on Fig. 2, the efficiency of the excitation of the second SLR in the ITO substrate is lower than that of the SLR in the organic medium. Thereby, we can conclude that the collective coupling of the LSPR modes in these structures occurs preferentially in the organic medium.

Finally, for the third zone $(p > 430\,{\text{nm}})$, we observe that the resonance peak of the UB becomes thinner (FWHM $= 20.4\,{\text{nm}}$) with an SLR-like behavior whereas the peak of the LB becomes broader (FWHM $= \,51\,{\text{nm}}$) with an LSPR-like behavior.

**Figure 3:**
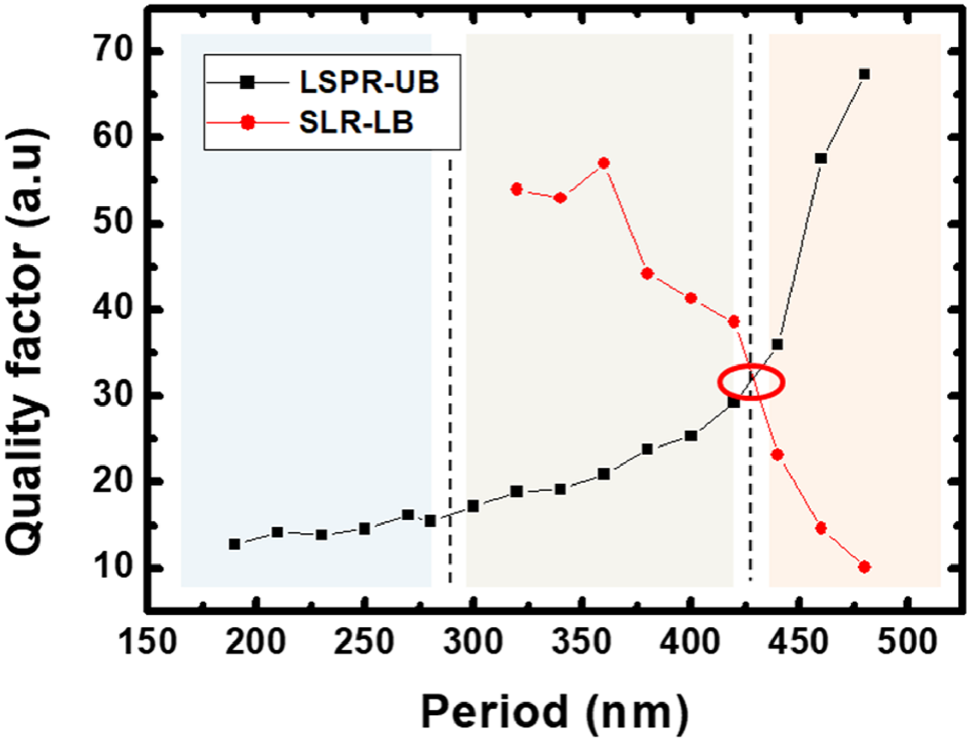
The quality factor associated to the principal resonance (LSPR-UB) and the excited narrow peak (SLR-LB) as a function of the period. Three zones can be distinguished *p* ≤ 280 nm, 280 nm < *p* ≤ 430 nm and *p* > 430 nm.

In order to pursue our analysis, we report on Fig. 3 the quality factor $Q$ of the main two peaks (LSPR-UB and SLR-LB) as a function of the grating period. The quality factor is related to the FWHM of the resonance peak, which is directly connected to the losses including metal damping, scattering and absorptions. It is very consistent to consider the evolution of $Q$ related to the three zones previously discussed. We can observe that the quality factor of the LSPR-UB mode increases versus the grating period, whereas it decreases in the case of the SLR-LB mode. One can, therefore, consider that the reduction of the quality factor associated to the SLR indicates a stronger damping effect, which might result from a higher localization of the field. On the other hand, the increase of the LSPR quality factor is synonymous of a reduction of damping as the period increases. Thus, we can speculate that this reversed behavior observed indicates that the energy is partially converted between the initial localized LSPR mode and the excited SLR mode.

More interestingly, the two curves intersect at the particular period of $430\,{\text{nm}}$ (quality factor of 32). For this period, and as shown on Fig. 2, the LSPR branch intersects the theoretical Rayleigh anomaly in the organic medium.

This analysis suggests that the quality factors of the LSPR-UB and SLR-LB branches is notably related to Rayleigh anomalies of the structure. Furthermore, we can speculate that the observed spectral changes are related to the existence of couplings that strongly alter the spectral characteristics of the main LSPR mode. The first zone ($p \le 280\,{\text{nm}})$, the LSPR-RA_org_ detuning is large which results in an array response dominated by the plasmonic feature^26^. The second zone ($280\,{\text{nm}} < p \le 430\,{\text{nm}}$) corresponds to a low detuning between the LSPR and the RA. In this period range the spatial and spectral overlap of the plasmonic mode with the diffractive grazing orders lead to the excitation of the hybrid lattice modes SLR. In fact, the induced SLR modes can be described as resulting from the coupling between a continuum energy state of the broad LSPR with the discrete state related to the Rayleigh anomaly^27^. In the third zone $(p > 430\,{\text{nm}})$, the initially LSPR mode penetrates the zone of the Rayleigh anomalies and an inverted trend is observed: the initial LSPR follows the dispersion of the Rayleigh anomalies in contrary to the peak of the SLR branch that exhibits a large resonance.

In other words, the obtained results emphasis the crucial role of the Rayleigh anomalies, which can be considered as the intermediary between individual plasmonic modes and collective photonic responses. In fact, the Rayleigh anomalies can be considered as a means of coupling between the LSPR and SLR modes. They allow the in-plane coupling between the dipolar moments of individual NPs^2^ and offer the possibility to excite mixed modes, the so-called hybrid SLR, which combine the high scattering cross-section of the LSPR modes, with the high quality factors of the diffractive orders to shape the spectral linewidth. In short, the Rayleigh Anomalies define the grating responses. This statement is in agreement with already reported results^14^.

### Nanoparticle grating: emitter coupling

This part of the work deals with the interaction between NP gratings and organic emitters. We are interested in the study of the influence of Ag-NP arrays on the emission of nearby organic emitters as a function of the grating period. We particularly investigate the role of the Rayleigh anomalies on the variation of the fluorescent lifetime and radiative emission patterns of the optically excited organic molecules. For that, we used an active organic layer of a guest host system of tris-(8-hydroxyquinoline) aluminum(Alq3) matrix doped with dichloromethane (DCM) emitters with a rate of 2% (Alq3:DCM) deposited on glass/ITO/grating substrates. These molecules have an intrinsic peak emission centered at 620 nm, in the spectral range of the resonances under study.

**Figure 4:**
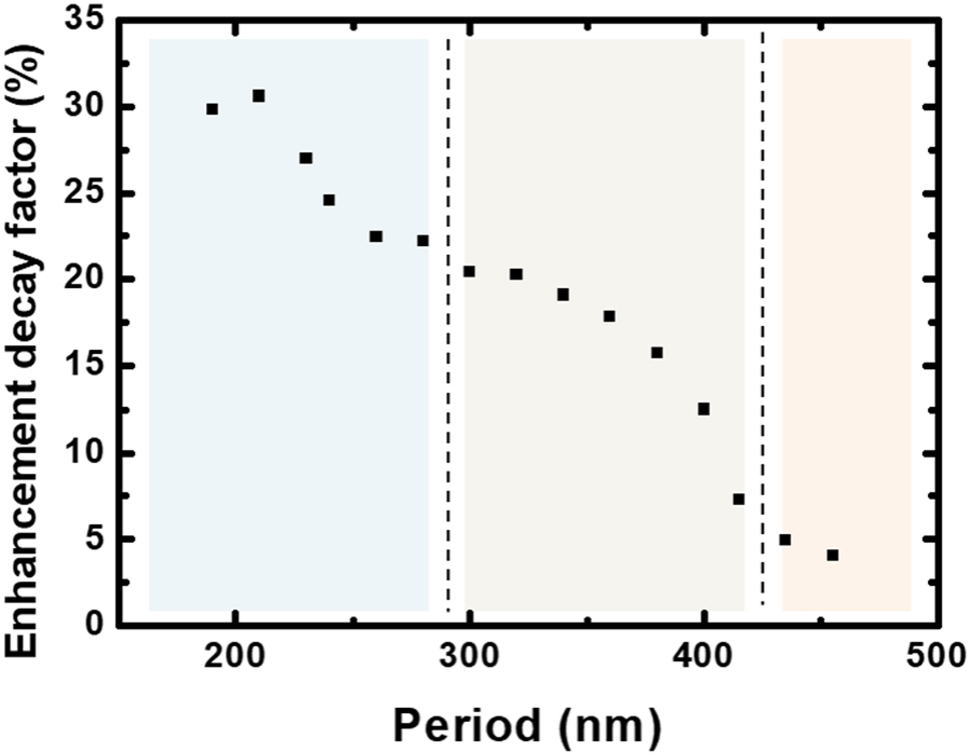
The evolution of the enhancement factors associated to the dye molecules Alq3: DCM as function of the period. Three zones can be distinguished *p* ≤ 280 nm, 280 nm < *p* ≤ 430 nm and *p* > 430 nm.

As a matter of fact, we carried out fluorescent lifetime measurements of the Alq3:DCM guest host system, deposited on NP gratings previously studied, using a time correlated single photon counting technique^28^. The Fig. 4 presents the obtained enhancement decay factor $\left( {1 - \frac{{\tau_{cav} }}{{\tau_{free} }}} \right)$ for Alq3:DCM system as a function of the grating period. Again, the same three zones discussed above can be considered.

This ratio gives the Purcell factor $F_{p}$ which quantifies the modification of the local density of states induced by the presence of a cavity. In fact, the modifications of the decay rates can be explained in terms of local density of states (DOS). Slight changes in the environment lead to a modification of the number of the accessible states in which the emission can occur^29^.

As a recall, the decay rate of an isolated emitter, in a free space, is given by^30^:

2 $$ \gamma_{free} = \frac{1}{{\tau_{free} }} = \frac{{\mu_{ij}^{2} \omega^{3} }}{{3\pi \hbar c^{3} }} $$

where $\tau_{free}$ is the radiative lifetime (without the plasmonic structure) of the free space, $\mu_{ij}$ the transition dipole moment and $\omega$ the frequency of the emitted light. The presence of a plasmonic structure in the vicinity of the emitter modifies the decay rate as^30^:

3 $$ \gamma_{cav} = \frac{1}{{\tau_{cav} }} = \beta^{2} \frac{{2\mu_{ij}^{2} }}{{\varepsilon_{0} \hbar }}\frac{Q}{V} $$

$$\tau_{cav}$$ is the radiative lifetime in the presence of the plasmonic structure, $$Q $$ the quality factor, $$V$$ the mode volume,$$ \beta$$ a factor that take into account the orientations of the dipole moments.

The enhancement factor is a good indicator of the dominant effect produced by the NPs on the emission process of the organic molecules. Thereby, regardless the period, in the presence of metallic gratings, the decay factor of the organic molecules is enhanced compared to that of the sample without NPs. More precisely, in the case of short grating periods with $p \le 280\,{\text{nm}}$, the enhancement factor reaches nearly 32% at $p = 210\,{\text{nm}}$. Whereas, in the range of periods $280\,{\text{nm}} < p \le 430\,{\text{nm}}$ the enhancement factor decreases to $\sim \,17\%$ and finally on the third period range with $p > 430\,{\text{nm}}$ the factor decreases to $\sim 4\%$ at $p = 455\,{\text{nm}}$.

Thus, it is suggested that NP-gratings dominated by the LSPR response (in the case of short periodicities and high LSPR-RA detuning) strongly modify the dynamics of the decay of the emitters. The obtained results are in agreement with other reports^17,18^. In contrast to that, for grating periods exhibiting SLR modes, the modification is lower which point out that the hybrid modes do not drastically affect the decay dynamics of the organic molecules.

These results emphasize that the Rayleigh anomalies do not influence the decay of the molecules, which is a near field process. Thereby, the structures with dominant LSPR responses locally modify the density of state (DOS) by adding new vacant states. The NP arrays act as a set of uncorrelated metallic nano-cavities that provide new decaying channels in their near field zones: the small modal volumes associated to the highly confined LSPR modes ensure high decay rates for the coupled emitters. Therefore, these latter can efficiently radiate into the LSPR modes that owns to faster decay dynamics and reduced lifetimes.

Besides, it is worth noting that in a guest–host system the emission results from a Förster energy transfer between the Alq3 donor molecules and DCM acceptor molecules^31^. It has been reported that the LSPR effect can enhance the transfer mechanism when the Plasmon resonance is located on the overlap range between the spectra of the emission of the donor and that of the acceptor absorption. However, in this study the LSPR is located at 670 nm, far from the emission spectrum of the Alq3 centered at 540 nm and the absorption spectrum of the DCM molecules occurring around 470 nm. Furthermore, although the hybrid SLR modes appear in this wavelength range, no particular effect is obtained on the emission lifetimes for this grating regime.

At this stage of the discussion, it is important to indicate that reduced lifetime does not necessary means an increase of the number of the emitted photons. In fact, the presence of the plasmonic structure provides two additional decay path associated to two competing processes: the first one is the radiative decay due to the coupling of the excited states to the bright LSPR modes of the Ag-NPs, and the second process is related to the quenching of the excited states mainly caused by coupling into high order plasmons modes (dark modes). These phenomena very likely determine the overall emission features. Another additional decay channel related to the presence of the SLR modes should be considered.

To go further in our study, we concentrate on the investigation of the emission pattern versus the grating period.

### Study of the radiation pattern

**Figure 5:**
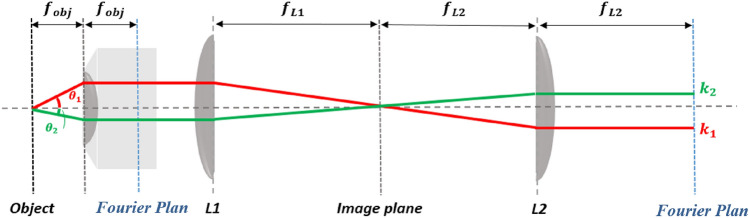
A scheme of the Fourier imaging setup.

Here, our goal is to gain more insight on the impact of the Rayleigh anomalies on the emission properties of the molecules, we studied the radiation pattern of the previous samples by using Fourier imaging technique^32^. A scheme of the setup is presented in the Fig. 5.

**Figure 6:**
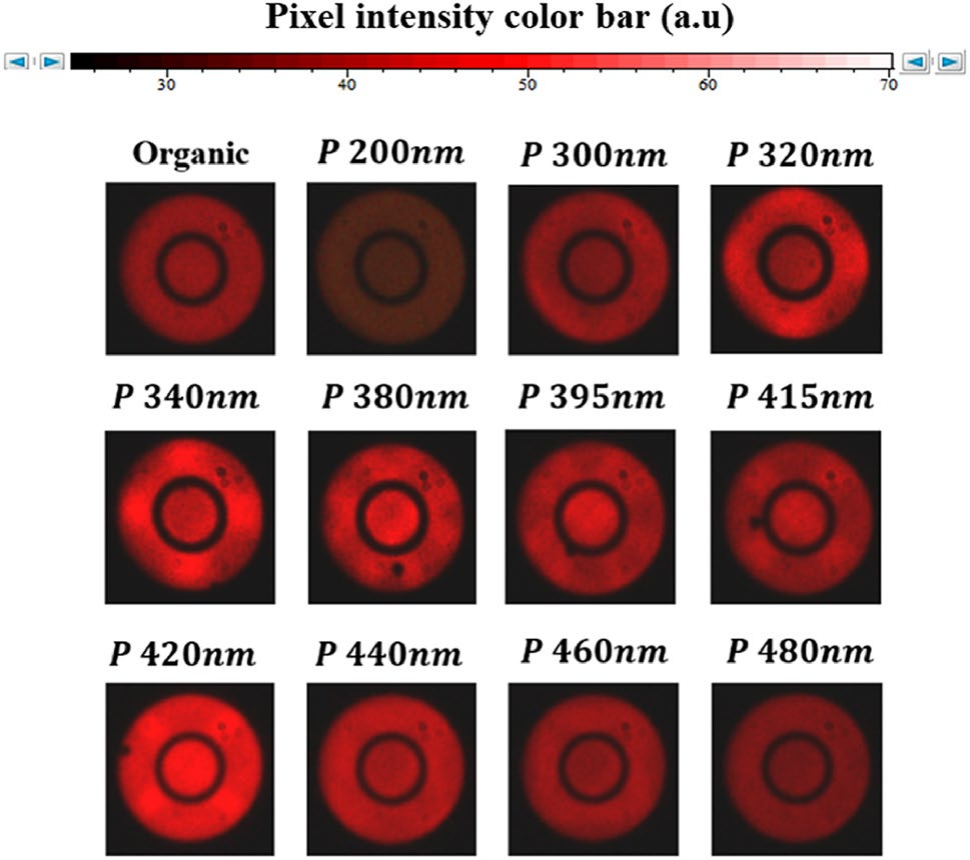
Fourier imaging of organic molecules optically pumped on top of metallic nanoparticles arrays of various periods.

The performed measurements are shown in Fig. 6. It should be noted that the presence of black ring in the images below is a specificity of the used objective which allows a phase contrast characterization.

We notice that for the sample without NP array, the emission pattern is uniform and does not exhibit any particular directionality as expected for dispersed emitters on top of a plane surface. A similar behavior is observed in the case of molecules on top of the grating with $p = 200\,{\text{nm}}.$ However, the emission intensity of this latter is clearly lowered compared to the sample without grating. As mentioned in the previous section, at short periodicities the grating modes are dominated by the LSPR feature. Thereby, the reduction of the emitted photons can be attributed to the damping process associated to the localized feature of the LSPR modes.

Up to a period of $p \sim 300\,{\text{nm}}$ the emission shows high intensities at well-defined directions that strongly depend on the grating period. We observe appearance of lobes. These features result from the geometrical arrangement of the NPs in the gratings^33,34^. They are diffracted orders associated to the in-plane wave-vectors $\vec{k}_{d, / / }$ that can be studied using the well-known diffraction condition of a 2D grating:

4 $$ \vec{k}_{d, / / } = \vec{k}_{inc, / / } + \vec{G} $$

$\vec{k}_{inc, / / }$ is the in plane incident wave vector and $\vec{G}$ the reciprocal lattice vector.

**Figure 7:**
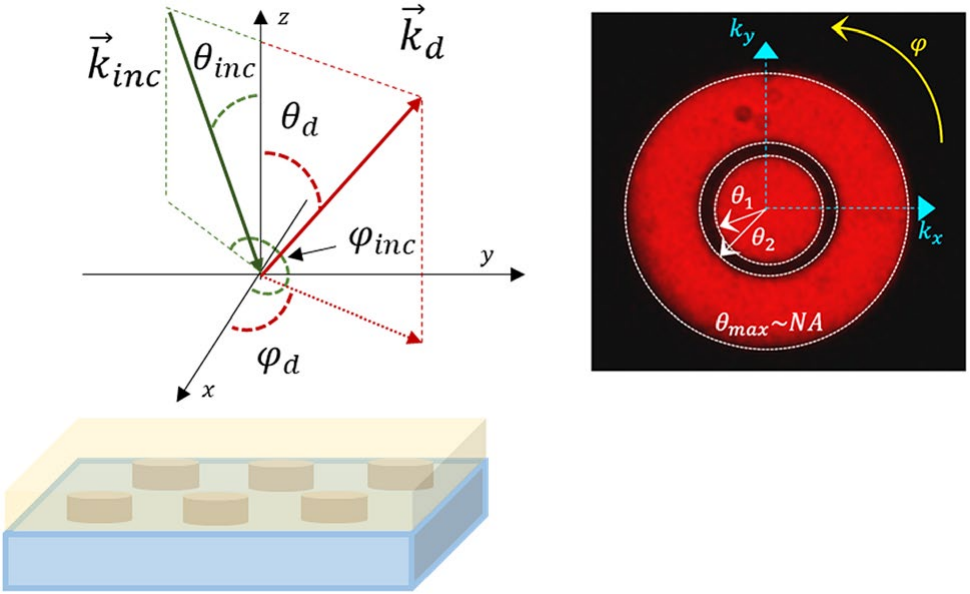
Scheme of the diffracted wave-vector and the typical recorded angular map.

The scheme of the diffracted wave-vector and the typical recorded angular map are presented in Fig. 7.

For periods ranging from $p \sim 300$ to $340\,{\text{nm}},$ we observe four lobes on the emission maps that correspond to the first diffracted orders $\left( { + 1;0} \right)$, $\left( {0; + 1} \right)$, $\left( { - 1;0} \right)$ and $\left( {0; - 1} \right)$. These orders experience the same evolutions when the period increases, which is the result of the square symmetry of the gratings. Therefore, we will focus on the study of a single order $\left( { + 1;0} \right)$.

The components of the diffracted in plane wave vectors associated to this order are given by the following equations:

5 $$ k_{d / / ,x} = \frac{2\pi }{\lambda }n_{d} \sin \theta_{d} \cos \varphi_{d} = \frac{2\pi }{\lambda }n_{inc} \sin \theta_{inc} \cos \varphi_{inc} + \frac{2\pi }{p} $$

6 $$ k_{d / / ,y} = \frac{2\pi }{\lambda }n_{d} \sin \theta_{d} \sin \varphi_{d} = \frac{2\pi }{\lambda }n_{inc} \sin \theta_{inc} \sin \varphi_{inc} $$

$$\lambda$$ is the emission wavelength of the Alq3:DCM molecules, $$n_{inc}$$ and $$n_{d} $$ are the refractive index associated to the incident and diffraction medium, $$p$$ is the period of the grating, $$\theta_{inc}$$ and $$\varphi_{inc}$$ are the out of plane and the in plane incident angles and $$\theta_{d}$$ and $$\varphi_{d}$$ are the out of plane and in plane diffracted angles, respectively.

Because $n_{inc} = n_{d} = 1,7$ the refractive index of the organic medium; we rewrite the system associated to the $\left( { + 1;0} \right)$ order as:

7 $$ \sin \theta_{d} \cos \varphi_{d} = \sin \theta_{inc} \cos \varphi_{inc} + \frac{\lambda }{{pn_{d} }} $$

8 $$ \sin \theta_{d} \sin \varphi_{d} = \sin \theta_{inc} \sin \varphi_{inc} $$

For this study, we also distinguish three sub-domains of periodicities to describe the evolution of the emission pattern of the studied samples. The first one corresponds to the periods ranging from $p \sim 300$ to $340\,{\text{nm}}$ with the appearance of four lobes on the maps. The second one is defined from *p* = 380–420 nm and show a strong modification of the intensity distribution in the plane of the gratings. And finally, a third domain associated to $p \ge 440\,{\text{nm}},$ which is characterized by a significant lowering of the directional features.

Initially, on the first domain, the $\left( { + 1;0} \right)$ mode show an important variation of the diffracted angle $\theta_{d}$ mainly around the in-plane direction $\varphi_{d} \sim 0$. We note that in this case, the gratings have periods $> \lambda /n_{d}$ ; with $\lambda /n_{d} \sim 358nm$ corresponding to the excitation of $\left( { + 1;0} \right)$ Rayleigh anomalies at $\varphi_{d} = 0$ under a normal incidence $\theta_{inc} = 0$.

In the second domain, the condition $p < \lambda /n_{d}$ is verified. We observe a strong variation of the in-plane diffracted angle $\varphi_{d}$ and the emergence of two lobes for each order. In particular, at a fixed $\theta_{d}$, the increase of the period leads to a higher separation between these lobes at well-defined angles $\pm \varphi_{d}$.

The transition between these two grating domains, from $340$ to $380\,{\text{nm}}$, occurs at $p = \lambda /n_{d} \sim 358\,{\text{nm}}$, which related to the condition of excitation to the $\left( { + 1;0} \right)$ Rayleigh anomalies.

From these results, one can conclude that the Rayleigh anomalies are at the origin of the observed spatial features. We believe that Rayleigh anomalies impose the excitation condition, which can be given by the following equation:

9 $$ \left| {\sin \theta_{inc} \cos \varphi_{inc} } \right| = 1 - \frac{\lambda }{{pn_{d} }} $$

For instance, we report in the Table 1 the experimental in plane diffracted angles $\varphi_{d,exp}$ compared to those calculated using the previous equation $\varphi_{d,th}$.

**Table 1:** Diffracted experimental and theoretical in plane angles as a function of the grating period.

Period (nm)	$\varphi_{d,\exp } \left(^\circ \right)$	$\varphi_{d,th} \left(^\circ \right)$
380	± 26	± 27.3
395	± 35	± 35.2
415	± 43	± 43.2
420	± 45	± 44.9

A very good agreement is obtained between the theoretical and experimental data, which confirms the validity of the previous equation and sustain our analysis.

In particular, these results show that periodic metallic structures are not equivalent to conventional gratings. Under specific conditions, they can exhibit diffraction modes. More precisely, they show that Rayleigh anomalies are at the origin of the coupling mechanism between the NPs as they determine the diffraction direction by defining the excitation condition for this set of the metallic nanoparticles to act as a diffraction grating.

Finally, the third domain corresponds to periods $p \ge 440\,{\text{nm}}$. In this range, a significant lowering of the directional features is observed; the diffracted modes are strongly damped and for a further increase of the period, we find almost a uniform distribution. This behavior can be attributed to a reduced collective effect, which can be explained by the fact that in this period range, the Rayleigh anomalies are no more excited following the direction of $\varphi_{d} = 0$ and, thus, the previous condition is no more fulfilled. As a consequence, the absence of the Rayleigh anomalies leads to the disappearance of a collective coupling between the NPs. This confirms the important role of Rayleigh anomalies in the shaping of the directivity of the emission.

**Figure 8:**
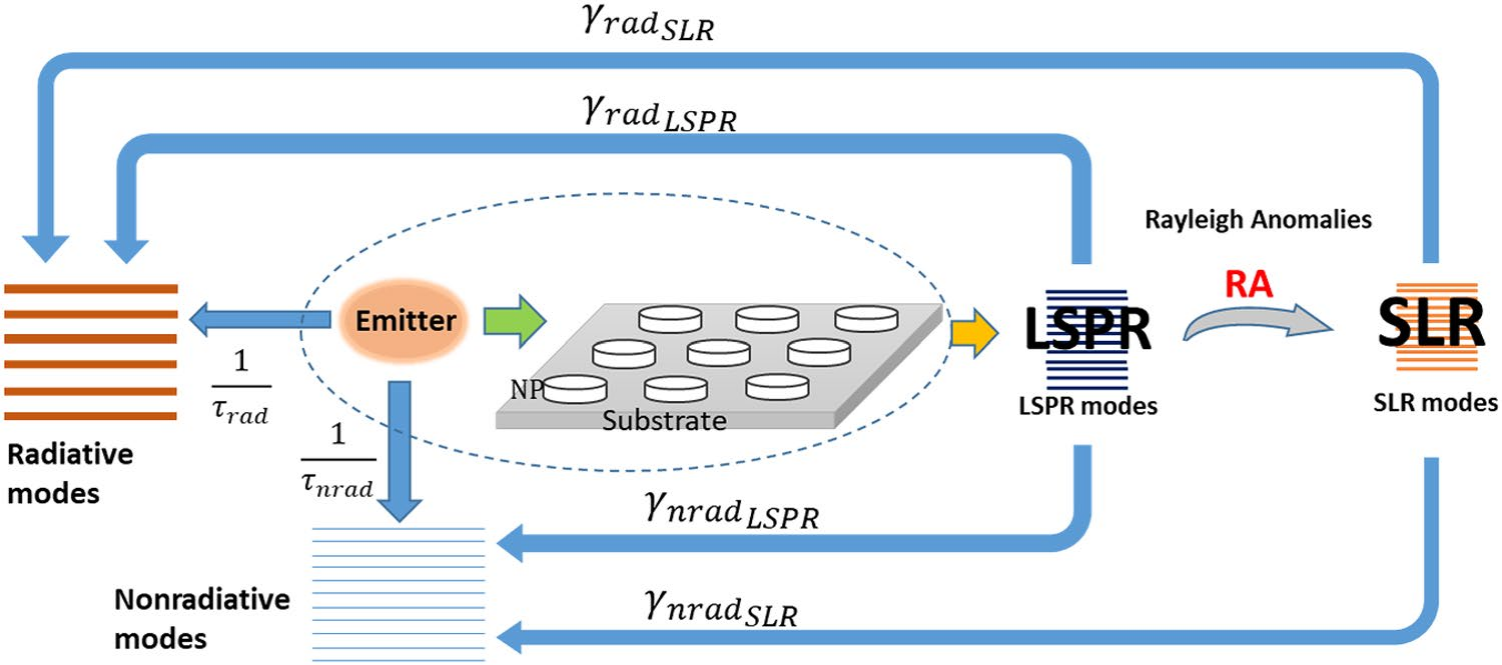
Illustration of the coupling between an emitter and a periodic grating of metallic NPs. Two mechanisms occur due to the presence of the LSPR and SLR connected by the Rayleigh anomalies.

Finally, from the previous discussions, we suggest the diagram presented in Fig. 8 as a general view of the different coupling mechanisms between emitters and NPs-grating. Indeed, similarly to what has been suggested for the coupling between emitters and an isolated NP^16^, we can consider the existence of two channels: LSPR and SLR. Both channels influence the radiative and non-radiative decay dynamics of the emitter. In particular, our results show that the LSPR effect contributes more efficiently to reduce the fluorescent lifetime of nearby emitters. However, the SLR modes that appears as the result of a coupling between the LSPR and the Rayleigh anomalies, strongly influence the directivity of the emission. Besides, the enhancement of the emitted power can be attributed to the combination of two effects: an enhanced plasmonic electrical field and the improvement of the spatial coherence of the emitted light by the excitation of diffractive modes. Furthermore, these effects are fully generated by the presence of Rayleigh anomalies in the spectral range of the main LSPR resonance.

To conclude, we report the investigation of Ag-ordered nanoparticles of square lattice and their influence on the optical properties of organic emitters under optical excitation. We particularly point-out the existence of two regimes: that of short periods with a dominant localized LSPR modes and the that of long periods resulting from the excitation of collective SLR modes. The transition from a regime to another is determined by the presence of the Rayleigh anomalies.

We study the coupling between the NP gratings and organic emitters by performing lifetime measurements and emission pattern characterizations. The obtained results emphasis the crucial role of the Rayleigh anomalies, which can be considered as the intermediary between individual plasmonic modes and collective photonic responses. In fact, the Rayleigh anomalies can be considered as a means of coupling between NPs as they define the excitation condition for which these NPs can coherently diffract the incident light.

Therefore, using metallic nanoparticle gratings permit to enhance, mold and reshape the emission of organic emitters with controllable spectral and spatial features, which is particularly crucial to develop efficient organic devices. Our work continues by considering the same phenomena in the case of organic light emitting diodes (OLED) and, thus, the influence of electrical pumping of such systems.

## Materials and methods

### Electronic beam Lithography

The technique is a based on the use of a focused beam of electrons to finely scan a substrate covered by an electron-sensitive resist to create various patterns^34^. The focused beam changes the solubility of the film which enables a selective removal of the resist. The choice of the technique is due to the fact that it provides a high degree of control and allows a precise fabrication of nano structured surfaces with desired size, shape, particle orientation and inter-particle distances well below the diffraction limit of the light.

### Extinction measurements

The obtained periodic structures have been optically characterized by performing transmission measurements using a microscope coupled to a 2000 Ocean Optics spectrometer. The samples have been excited using supercontinuum white light laser, the transmitted responses have been collected by an objective lens of 0.6 NA coupled to spectrometer working on the visible range.

The extinction map is obtained by considering the ratio of the transmission spectrum of the reference structure $T_{0}$ (Glass/ITO/Organic layer) to those associated to the metallic gratings $T$. In this case, the maximum on extinction map (given by the red color) corresponds to a loss of the signal that is only due to the presence of the gratings. In fact, the reflection and scattering losses associated to the sample are already contained within the transmission of the reference sample $T_{0}$.

### Time-correlated single photon counting (TCSPC)

The radiative lifetime of excited dyes on top of the different lattices have been measured by using time correlated single photon counting technique^28^. The concept is based on a repetitive timed registration of single photons by multiple cycles of excitation and emission. The decay profile is reconstructed by collecting the single photon events over many cycles. The measurements are obtained as histogram representing the photon arrivals per time with the time reference corresponding to the excitation pulse.

In this study, we use a laser beam pulse with an excitation wavelength at 405 nm and a repetition rate of 1 MHz to excite the Alq3 matrix.

### Fourier imaging

This technique^32^ allows the characterization of the emission directivity as it provides the angular power distribution. The setup consists of using a pump laser of 405 nm focused on the sample using an objective lens of $NA = 0.6$. The emitted light from the sample is shaped using a set of optical lenses and recorded using a charge coupled device (CCD), which is precisely positioned to give the image of the field intensity at the back focal plane of the objective lens.

The obtained maps give the angular power distribution in the polar coordinates $\left( {\theta , \varphi } \right)$. The radius gives the elevation angle $\theta$, whereas $\varphi$ represents the azimuthal angle.The numerical aperture $\left( {\theta_{\max } \sim 36^\circ } \right)$ of the objective represents the maximum angle that can be detected on the CCD. Besides, we must point out the appearance of a dark ring in all the images.

This is a characteristic of the entrance lens due to the dimensions of the objective used that allows a phase contrast characterization, which blocks the light in the crown delimited by the angles $\theta_{1} \sim 14^\circ$ and $\theta_{2} \sim 20^\circ$.

In order to calibrate our setup, we used a small source (typically a compact red laser diode illuminating a pinhole of 5 µm of diameter) placed on a goniometer and considered as the excited sample. For each defined angle at the entrance of the objective lens, we determine the pixel location at the camera placed at the Fourier plan. In this way, we were able to precisely define the different angles on the maps.

### Spectral and lifetime measurements

A typical normalized emission spectrum of Alq3:DCM organic layer on top of a glass/ITO substrate without NPs is presented below.
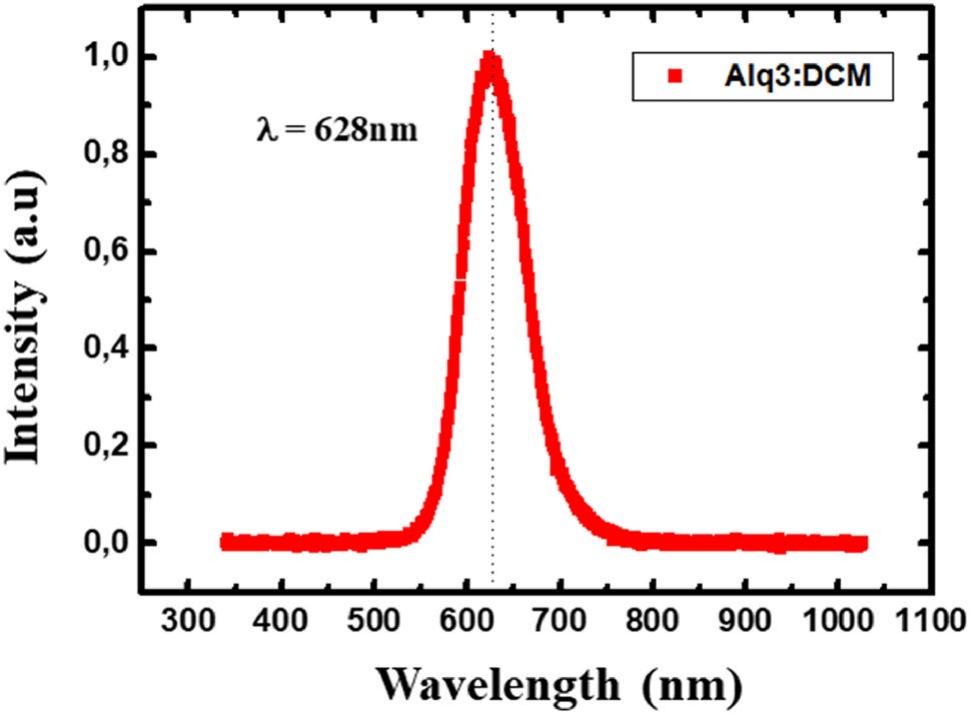


The decay lifetime measurement curves with and without nanoparticle gratings are displayed below. The measurements have been performed using the same experimental conditions and a pump source of 405 nm at a frequency of 1 MHz. For the detection, we used a spatial filter operating at 520 nm and a single photon counter to collect the emitted photons. The calculated lifetimes have been obtained by fitting the curves assuming a single exponential decay.
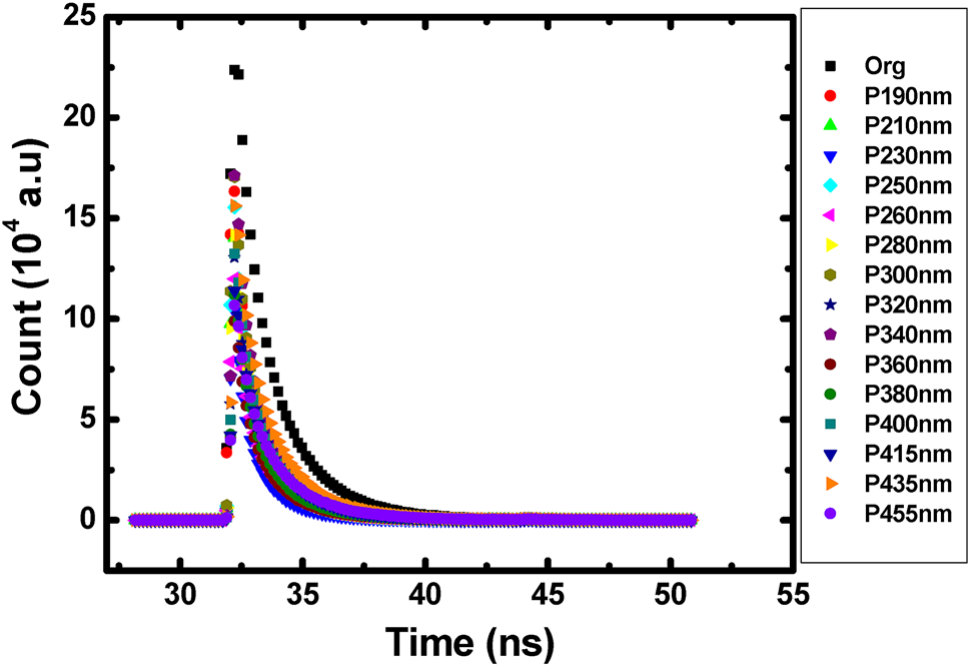


## References

[1] Salerno M, Krenn JR, Hohenau A, Ditlbacher H, Schider G, Leitner A, Aussenegg FR (2004). The optical near-field of gold nanoparticle chains. Optics Commun..

[2] Zhou W, Odom TW (2011). Tunable subradiant lattice plasmons by out-of-plane dipolar interactions. Nat. Nanotechnol..

[3] Evlyukhin B, Reinhardt C, Zywietz U, Chichkov BN (2012). Collective resonances in metal nanoparticle arrays with dipole-quadrupole interactions. Phys. Rev. B.

[4] Lamprecht B, Schider G, Lechner RT, Ditlbacher H, Krenn JR, Leitner A, Aussenegg FR (2000). Metal nanoparticle gratings: Influence of dipolar particle interaction on the plasmon resonance. Phys. Rev. Lett..

[5] Jain PK, El-Sayed MA (2010). Plasmonic coupling in noble metal nanostructures. Chem. Phys. Lett..

[6] Chen HY, He CL, Wang CY, Lin MH, Mitsui D, Eguchi M, Teranishi T, Gwo S (2011). Far-field optical imaging of a linear array of coupled gold nanocubes: Direct visualization of dark plasmon propagating modes. Am. Chem. Soc..

[7] Wang W, Ramezani M, Väkeväinen AI, Törmä P, Rivas JG, Odom TW (2018). The rich photonic world of plasmonic nanoparticle arrays. Mater. Today.

[8] Zhang Y, Demesy G, Haggui M, Gerard D, Béal J, Dodson S, Xiong Q, Plain J, Bonod N, Bachelot R (2017). Nanoscale switching of near-infrared hot spots in plasmonic oligomers probed by two-photon absorption in photopolymes. ACS Photon..

[9] Liang FX, Ge CW, Zhang TF, Xie WJ, Zhang DY, Zou YF, Zheng K, Luo LB (2017). Plasmonic Hollow gold nanoparticles induces high-performance BiS2S3 nanoribbon photodetector. Nanophotonics..

[10] Garcia MA (2011). Surface plasmons in metallic nanoparticles: Fundamentals and applications. J. Phys. D Appl. Phys..

[11] Maradudin A, Simonsen I, Polancoand J, Fitzgerald RM (2016). Rayleigh and Wood anomalies in the diffraction of light from a perfectly conducting reflection grating. J. Opt..

[12] Hessel A, Oliner AA (1965). A new theory of wood’s anomalies on optical gratings. Appl. Opt..

[13] Khlopin D, Laux F, Wardley WP, Martin J, Wurtz GA, Plain J, Gérard D (2017). Lattice modes and plasmonic linewidth engineering in gold and aluminum nanoparticle arrays. J. Opt. Soc. Am. B..

[14] Vecchi G, Giannini V, Gómez Rivas J (2009). Shaping the fluorescent emission by lattice resonances in plasmonic crystals of nanoantennas. Phys. Rev. Lett..

[15] Giannini V, Vecchi G, Gómez Rivas J (2010). Lighting up multipolar surface plasmon polaritons by collective resonances in arrays of nanoantennas. Phys. Rev. Lett..

[16] Khurgin JB, Sun G (2009). Enhancement of optical properties of nanoscaled objects by metal nanoparticles. J. Opt. Soc. Am. B..

[17] Neal TD, Okamoto K, Scherer A (2005). Surface plasmon enhanced emission from dye doped polymer layers. Opt. Express.

[18] Malicka J, Gryczynski I, Maliwal BP, Fang J, Lakowicz JR (2003). Fluorescence spectral properties of cyanine dye labeled DNA near metallic silver particles. Biopolymers.

[19] des Francs Colas G, Derom S, Vincent R, Bouhelier A, Dereux A (2012). Mie plasmons: Modes volumes, quality factors, and coupling strengths (purcell factor) to a dipolar emitter. Int. J. Opt..

[20] Bittona O, NathGuptaa S, Haran G (2019). Quantum dot plasmonics: From weak to strong coupling. Nanophotonics..

[21] Muskens OL, Giannini V, Sánchez-Gil JA, Rivas JG (2007). Strong enhancement of the radiative decay rate of emitters by single plasmonic nanoantennas. Nano Lett..

[22] Aouani H, Mahboub O, Devaux E, Rigneault H, Ebbesen TW, Wenger J (2011). Plasmonic antennas for directional sorting of fluorescence emission. Nano Lett..

[23] Aouani H, Mahboub O, Bonod N, Devaux E, Popov E, Rigneault H, Wenger J (2011). Bright unidirectional fluorescence emission of molecules in a nanoaperture with plasmonic corrugations. Nano Lett..

[24] Nikitin AG, Kabashin AV, Dallaporta H (2012). Plasmonic resonances in diffractive arrays of gold nanoantennas: Near and far field effects. Opt. Express.

[25] West PR, Ishii S, Naik GV, Emani NK, Shalaev VM, Boltasseva A (2010). Searching for better plasmonic materials. Laser Photon. Rev..

[26] Rodriguez SRK, Abass A, Maes B, Janssen OTA, Vecchi G, Gómez Rivas J (2011). Coupling bright and dark plasmonic lattice resonances. Phys. Rev. X..

[27] Miroshnichenko AE, Flachand S, Kivshar YS (2010). Fano resonances in nanoscale structures. Rev. Mod. Phys..

[28] Wahl, M. Time correlated single Photon counting. *PicoQuant GmbH* ; https://www.picoquant.com/images/uploads/page/files/7253/technote_tcspc.pdf (2014).

[29] Shahbazyan TV (2018). Spontaneous decay of a quantum emitter near a plasmonic nanostructure. Phys. Rev. B.

[30] Giannini V, Fernández-Domínguez AI, Heck SC, Maier SA (2011). Plasmonic nanoantennas: Fundamentals and their use in controlling the radiative properties of nanoemitters. Chem. Rev..

[31] Ramos-Ortiz G, Oki Y, Domercq B, Kippelen B (2002). Förster energy transfer from a fluorescent dye to a phosphorescent dopant: A concentration and intensity study. Phys. Chem. Chem. Phys..

[32] Vasista, A. B., Sharma, D. K. and Kumar, GV. P. Fourier plane optical microscopy and spectroscopy. *Digital Encyclopedia of Applied Physics*. 1–44; https://arxiv.org/abs/1806.08280 (2019).

[33] Lozano G, Grzela G, Verschuuren MA, Ramezani M, Rivas JG (2014). Tailor-made directional emission in nanoimprinted plasmonic-based light-emitting devices. Nanoscale.

[34] Chen Y (2015). Nanofabrication by electron beam lithography and its applications: A review. Microelectron. Eng..

